# Chemical Composition, Total Phenolic Content, and Antioxidant Activities of the Essential Oils of the Leaves and Fruit Pulp of *Annona muricata* L. (Soursop) from Ghana

**DOI:** 10.1155/2019/4164576

**Published:** 2019-09-02

**Authors:** Joseph Nana Gyesi, Rita Opoku, Lawrence Sheringham Borquaye

**Affiliations:** ^1^Department of Chemistry, Kwame Nkrumah University of Science and Technology, Kumasi, Ghana; ^2^Central Laboratory, Kwame Nkrumah University of Science and Technology, Kumasi, Ghana

## Abstract

*Annona muricata*, also called soursop, is widespread in many tropical countries, and various parts of the plant have been shown to possess very good pharmacological properties. This work evaluated the chemical composition and antioxidant activities of essential oils obtained from the fruit pulp and leaves of soursop. Essential oils were obtained via hydrodistillation and characterized by gas chromatography-mass spectrometry. Antioxidant potential was evaluated via the phosphomolybdenum, hydrogen peroxide scavenging, and 1,1-diphenyl-2-picrylhydrazyl (DPPH) free radical scavenging assays. In the leaf essential oil, a total of 31 compounds were identified with *δ*-cadinene (22.58%) and *α*-muurolene (10.64%) being the most abundant. Thirty-two compounds were identified in the fruit pulp essential oil with Ç-sitosterol (19.82%) and 2-hydroxy-1-(hydroxymethyl) ethyl ester (13.48%) being present in high amounts. Both essential oils showed very good total antioxidant capacities (49.03 gAAE/100 g and 50.88 gAAE/100 g for fruit pulp and leaf essential oils, respectively). The IC_50_ values from the DPPH assay were 244.8 ± 3.2 *μ*g/mL for leaf essential oil and 512 ± 5.1 *μ*g/mL for the fruit pulp essential oil. At 1 mg/mL, hydrogen peroxide scavenged was below 50% for both leaf and fruit pulp essential oils, indicating moderate activity. These results suggest possible application of the essential oils of *Annona muricata* in food preservation and processing.

## 1. Introduction

Antioxidants have garnered a lot of attention in recent times due to their potential in the prevention and treatment of radical-mediated diseases. Reactive oxygen species (ROS) and reactive nitrogen species (RNS) provide oxidative radicals which interact with and damage macromolecules such as proteins, lipids, and DNA [1–3]. Oxidative damage to these macromolecules has been implicated in a number of human pathological processes such as diabetes, pulmonary dysfunction, atherosclerosis, and neurological diseases [4–6]. Lipid oxidation also plays a critical role in the food industry. Reduction in food quality and loss of nutritional value are associated with the oxidative degradation of lipids [7]. To ameliorate the effects of radicals, various compounds have been explored. Compounds such as butylated hydroxyanisole (BHA) or butylhydroxytoluene (BHT) are commonly used in the food industry as antioxidants [8]. These synthetic compounds are, however, suspected to have harmful effects on human health, and their use is therefore discouraged [9, 10]. Some of the synthetic antioxidants (such as *α*-tocopherol and BHT) exhibit poor activities in complex food systems [11] and foods enriched with long chain omega-3 fatty acids [12]. Natural antioxidants with low toxicities have therefore been the focus of many research works. One class of natural products that have shown promise in this regard is essential oils [13].

Essential oils are aromatic, oily liquids made up of mixtures of volatile compounds obtained usually via hydrodistillation, steam distillation, dry distillation, or a mechanical process without the application of heat from a plant or a plant part. They usually possess a strong odor, are seldom colored, and are generally lower in density relative to water. Essential oils have been isolated from all plant organs such as flowers, buds, seeds, leaves, twigs, bark, herbs, wood, fruits, and root [14, 15]. The use of essential oils for their beneficial human properties has been in existence since antiquity and is documented in primordial literature [16, 17]. Some of these properties of essential oils such as anti-inflammatory, antimicrobial, and antioxidant have been verified in the recent scientific literature [14, 18–20]. The oxidative stability of essential oils stems largely from the enormous diversity of compounds that constitute these oils [18]. Several essential oils are known to possess very good antioxidant capabilities, and this can be utilized in the protection of other substances, like food and animal feed preservation. Animal products (egg, meat, and fat) with improved dietary value, better oxidative stability, and longer shelf life may be obtained by using essential oils as additives in feedstuffs for farm animals [21].

*Annona muricata* L. (Annonaceae), also called soursop, is a tropical plant found in parts of the Americas, Asia, Australia, and Africa. It possesses thick leaves that are shiny on the upper side. The leaves are obovate, oblate, and acuminate to varying degrees. The soursop fruit is dark green, prickly, and ovoid with juicy, acidic, whitish, and aromatic pulp [22, 23]. The use of *Annona muricata* in traditional medicine is well documented. Ethnobotanical studies have described the plant as being used to treat various disease conditions like bacterial infections, fever, respiratory and skin illness, diabetes, internal and external parasites, hypertension, inflammation, pain, and cancer [22]. Extracts of the plant have been shown to possess antimicrobial, anti-inflammatory, antiprotozoan, antioxidant, and antitumor characteristics. *In vivo* studies of the plant extracts has revealed wound healing, antiulceric, hepatoprotective, anxiolytic, contraceptive, antistress, hypoglycemic, anti-inflammatory, antitumoral, and anti-icteric activities. Over 200 compounds have been isolated from the plant with most being alkaloids, phenols, and acetogenins [22]. Some acetogenins and alkaloids isolated from the plant have shown neurotoxicity [22–24].

Essential oils from various parts of *Annona muricata* have been the subject of a number of investigations. Essential oils have been isolated from different organs of the plant such as fruit pulp, leaves, and fruit peel as well as plants from different locations in Africa, Asia, and the Americas [25–37]. Interestingly, for the same plant part, differences in the chemical composition of the essential oils have been observed. This motivated this work on *Annona muricata* from Ghana. The present work describes the composition of essential oils from the leaves and fruit pulp of Ghanaian cultivars of *Annona muricata*. In addition, the antioxidant activities of the essential oils were evaluated.

## 2. Methods

### 2.1. Plant Material

Fresh fruits of *Annona muricata* L. were purchased from the local market in Kumasi, Ghana. Fresh leaves were collected from a farm at Ayeduase in Kumasi. Plant identification and authentication were carried out at the Herbarium Section of the Department of Theoretical and Applied Biology, Kwame Nkrumah University of Science and Technology (KNUST), Kumasi.

### 2.2. Isolation of Volatile Compounds

The pulp of the fresh fruit was separated from the peel and seed and 680 g of the pulp placed in a hydrodistillation apparatus. The setup for hydrodistillation consisted of a modified Clavenger glassware, a condenser, a round bottom flask, a heating mantle, and a receiving flask as described elsewhere. After 4 hours of distillation, 780 mg (0.11%) of essential oil was obtained. Similarly, 120 g of fresh leaves was placed in the hydrodistillation apparatus, and after 4 hours of distillation, 800 mg (∼0.67%) of essential oil was obtained.

### 2.3. Gas Chromatography Analysis

Gas chromatography-mass spectrometer (GC-MS) analysis of the essential oils from fruit pulp and leaves were performed using a gas chromatograph (PerkinElmer GC Clarus 580) interfaced to a PerkinElmer mass spectrometer (Clarus SQ 8 S) equipped with Elite-5MS (5% diphenyl/95% dimethyl poly siloxane) fused capillary column (30 × 0.25 mm ID × 0.25 *μ*m DF). At first, the oven temperature was maintained at 35°C for 2 minutes, and then it was ramped up to 250°C at a rate of 10°C/minute and finally to 320°C at a rate of 20°C/minute where it was held for 23 minutes. For mass spectrometer detection, an electron ionization system was operated in the electron impact mode. Helium was used as a carrier gas at a constant flow rate of 1 mL/minute, and an injection volume of 1 *μ*L was employed. The injector temperature was maintained at 250°C, and the ion-source temperature was kept at 150°C. Mass spectra were taken at 70 eV and a scan interval of 0.5 seconds over a mass range of 50 to 450 Da. The solvent delay was 0 to 4 minutes, and the total GC/MS run time was 50 minutes. Constituents were identified by comparison of their retention indices relative to *n*-alkanes and fragmentation pattern from mass spectra, which were compared to the mass spectra in the database of National Institute Standard and Technology (NIST) and published literature of spectral data whenever possible. The assigned compound names were made solely by using the similarity indices obtained from the Wiley and NIST libraries for the GC-MS system used and some published literature of spectral data. The relative percentages of the various constituents were expressed as percentages calculated by normalization of the peak area [38, 39].

### 2.4. Evaluation of Antioxidant Activities

Antioxidant potential of essential oils and hydrosols was evaluated by different *in vitro* assays; the phosphomolybdenum assay, hydrogen peroxide scavenging assay, and the 2,2-diphenyl-1-picrylhydrazyl (DPPH) radical scavenging assay. All chemicals used for the antioxidant activity evaluations were obtained from Sigma-Aldrich (St. Louis, MO, USA).

#### 2.4.1. Phosphomolybdenum (PM) Assay

The assay relies on the reduction of Mo (VI) to Mo (V) by the analyte of interest followed by the subsequent formation of a green phosphate/Mo (V) complex at acidic pH [40]. Five milliliters of the PM reagent (0.6 M sulphuric acid, 28 mM sodium phosphate, and 4 mM ammonium molybdate) was added to 0.5 mL of each test sample in a test tube. The test tubes were capped and shaken and then incubated at 95°C for 90 minutes. After samples had cooled to room temperature, absorbance was taken at 695 nm against a blank solution. Blank solution was made by replacing the test sample with solvent in the mixture and incubated under similar conditions. Ascorbic acid was used as the standard. Antioxidant capacity was expressed as equivalents of ascorbic acid with the following equation:

(1) $$\begin{array}{c} \text{TAC}=\frac{C\times \;V}{M}\times 100\;, \end{array}$$

where TAC is the total antioxidant capacity in gAAE/100 g of the test sample, *C* is the concentration of ascorbic acid (*μ*g/mL), *V* is the volume of the reaction mixture, and *M* is the mass of the essential oil in the reaction mixture.

#### 2.4.2. Hydrogen Peroxide Scavenging Assay

This method is based on the ability of 1,10-phenanthroline to form an orange complex with ferrous ion and the reduction of ferrous ion to ferric ion by hydrogen peroxide [41]. To 0.5 mL ferrous ammonium sulphate (1 mM) solution was added 3 mL of varying concentrations of essential oil (prepared in 5% DMSO) and 0.13 mL of 5 mM H_2_O_2_. The samples were incubated at room temperature away from light for 5 minutes. Thereafter, 3 mL 1,10-phenanthroline (1 mM) was added to each tube, mixed well and incubated again at room temperature for 10 minutes. The absorbance of each reaction mixture was taken at 510 nm using UV-Vis spectrophotometer. Water was used in place of essential oil for the blank solution. Percent hydrogen peroxide scavenged was calculated from the following equation

(2) $$\begin{array}{c} \text{\% hydrogen peroxide scavenged}=\;\frac{A_{\text{test}}}{A_{\text{control}}}\;\times \;100, \end{array}$$

where *A*_test_ is the absorbance of the test sample and *A*_control_ is the absorbance of the blank.

#### 2.4.3. DPPH Assay

The DPPH radical scavenging activity of the essential oils was evaluated using slightly modified standard methods. Essential oils of different concentrations were prepared in 5% DMSO. A solution of DPPH (0.1 mM) was prepared in methanol. To 40 *μ*L of essential oil was added 160 *μ*L of DPPH solution. The reaction mixture was thoroughly mixed together and incubated in the dark at room temperature for 30 minutes. Thereafter, the absorbance of the mixture was read at 517 nm. For the reaction blank, methanol was used in the place of essential oils. Ascorbic acid was used as the positive control. The percent DPPH radical scavenged was calculated from the following equation:

(3) $$\begin{array}{c} \text{\% DPPH radical scavenged}=\left(\frac{A_{\text{control}}-A_{\text{sample}}}{A_{\text{control}}}\right)\;\times \;100, \end{array}$$

where *A*_control_ is the absorbance of blank and *A*_sample_ is the absorbance of sample mixture [42, 43].

#### 2.4.4. Total Phenolic Content (Folin–Ciocalteu Method)

This method is based on the oxidation of phenolic groups by using Folin–Ciocalteu's reagent. To 5 mL of 10% Folin–Ciocalteu reagent was added 1 mL of sample solution of varying concentrations. The mixture was thoroughly mixed and allowed to stand for 5 minutes at room temperature. After this, 4 mL of 7% NaHCO_3_ was added slowly and the reaction mixture was allowed to stand in the dark for 30 minutes at room temperature. Absorbance readings were taken at 765 nm. The same procedure was repeated for all standard gallic acid solutions. The phenolic content was obtained from the gallic acid calibration curve and expressed as grams of gallic acid equivalent per 100 gram of dry weight of sample (gGAE/100 g) [39].

#### 2.4.5. Data Analysis

All experiments were conducted in triplicates and data presented as mean ± standard deviation. Statistical analysis was performed using GraphPad Prism 6.0 for Windows (GraphPad Software, San Diego, CA, USA). Where necessary, significance was evaluated using analysis of variance (ANOVA) followed by Sidak's multiple comparison test at *P* < 0.05.

## 3. Results

The essential oils from the leaf and fruit pulp of *Annona muricata* were obtained by hydrodistillation in a Clavenger-type set up. For the fruit pulp, an amber-colored oil, representing a yield of 0.11%, was obtained after concentration *in vacuo*. A light yellow oil was obtained from the hydrodistillation of fresh leaves of *Annona muricata*, with a yield of 0.67%.

**Figure 1:**
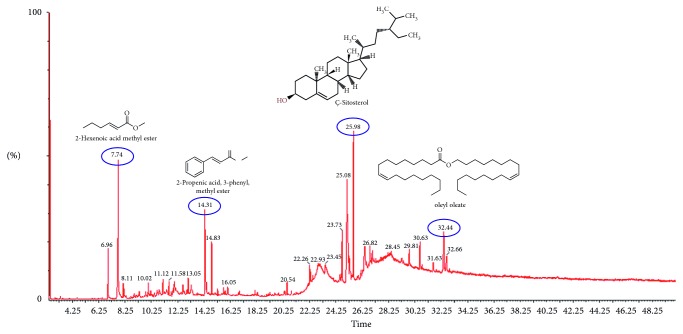
GC-MS spectra of the fruit pulp essential oil of *Annona muricata* and the chemical structure of some of the constituents.

**Figure 2:**
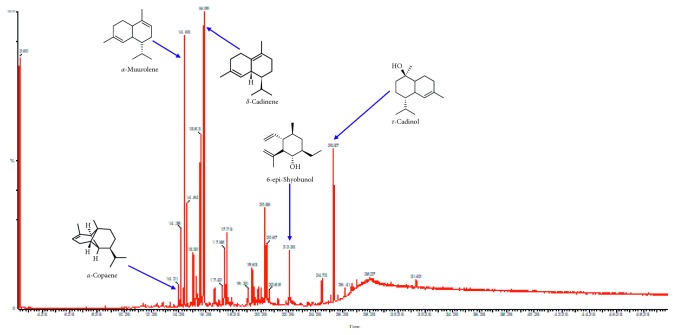
GC-MS spectra of the leaf essential oil of *Annona muricata* and the chemical structure of some of the constituents.

The total ion chromatogram (TIC) obtained from the GC-MS analysis of the essential oils are presented in Figure 1 (fruit pulp) and Figure 2 (leaf). For the leaf essential oil, 31 compounds representing 99.98% of the constituents were identified (Table 1), whereas 32 compounds, representing 99.99%, were identified in the fruit pulp essential oil (Table 2). There was marked difference in the chemical composition of the two oils. Whereas the leaf essential oils consisted largely of terpenes and terpenoids, the fruit pulp essential oils were made up of aliphatic compounds (acids, esters, and alcohols). The sesquiterpenes, *δ*-cadinene (22.58%), and *α*-muurolene (10.64%) were the most abundant compounds identified in the leaf essential oils. The diterpenoid, andrographolide, was also present in large amounts with a composition of 6.51%. *τ*-cadinol, ledene oxide (II), *α*-cardinol, and *β*-caryophyllene completed the top six most abundant compounds in the leaf essential oils. 3-(Octadecyloxy) propyl ester (5.57%) and octadecane (5.33%) were the most abundant aliphatic compounds present in the leaf essential oil. Gitoxigenin, a phytosterol, was also present. D-limonene and *α*-pinene, both monoterpenes, were also identified in the leaf essential oil. *α*-pinene was present in the lowest concentration, making up only 0.04% of the total composition.

The essential oil from the fruit pulp was made up of mainly aliphatic compounds and phytosterols. The most abundant compound was the phytosterol, *ς*-sitosterol, which made up 19.82% of the essential oil. Aliphatic compounds like 2-hydroxy-1-(hydroxymethyl) ethyl ester (13.48%), 2-hexenoic acid, methyl ester (10.27%), 2-propenoic acid, 3-phenyl-, methyl ester (8.67%), and 9-octadecanoic acid (4.59%) were also present in high amounts. Terpene compounds identified in the essential oil were squalene (a triterpene) and the sesquiterpenes, 4,5-di-epi-aristolochene and *β*-guaiene. Other phytosterols present were *α*-sitosterol (3.04%), *γ*-sitosterol (4.96%), and campesterol (2.19%). A number of straight-chain alkanes such as nonane, dodecane, and tridecane were also present in varying amounts. Phenylacetaldehyde was the only aldehyde identified in the essential oil from the fruit pulp of *Annona muricata*.

The antioxidant activities of the essential oils were determined using the phosphomolybdenum assay and DPPH and H_2_O_2_ scavenging assays. The total phenolic content of the oils was also determined. The total antioxidant capacity, as determined from the phosphomolybdenum assay, was 49.03 gAAE/100 g and 50.88 gAAE/100 g for fruit pulp and leaf essential oils, respectively (Table 3). There was no significant difference (*P* < 0.05) between the antioxidant capacities of both the leaf and fruit pulp essential oils. The leaf essential oil proved to be a better scavenger of the DPPH radical than the fruit pulp essential oil. The IC_50_ for the leaf essential oil was 244.8 ± 3.2 *μ*g/mL whereas that for the fruit pulp was 512 ± 5.1 *μ*g/mL (Table 4). At 1.0 mg/mL, the fruit pulp essential oil scavenged about 24% of H_2_O_2_ while the leaf essential oil scavenged about 32% of H_2_O_2_ at 1 mg/mL (Table 4). The leaf essential oil thus proved to be a better H_2_O_2_ scavenger than the fruit pulp essential oil. In terms of phenolic content, there were more phenolic compounds in the leaf essential oil than there was in the fruit pulp essential oil (Table 3).

## 4. Discussions

The yield of the essential oils obtained in this work was in a similar range to that obtained from *Annona muricata* by other researchers. In general, essential oil yields are less than 1%. The fruit pulp produced a slighter higher yield than the leaves in this study. Different plant organs produce different amounts/levels of essential oils, and this usually reflects the function of the oils in that plant organ.

The leaf essential oils were mainly terpenes, whereas the pulp essential oils were made up of aliphatic compounds and sterols, similar to other results in the literature [28–30, 33]. The most abundant component of the leaf essential oil was *δ*-cadinene. This compound has been identified in the leaf essential oils of *Annona muricata* from other locations as shown in Table 5 [27, 29, 32, 33]. Other works on the leaf essential oils of *Annona muricata* (Table 5) showed that *β*-caryophyllene was the major constituent [27, 29, 33]. Even though present in the leaf essential oil from Ghana, *β*-caryophyllene was not the most abundant constituent. Other sesquiterpenes and terpenoids such *α*-muurolene, *τ*-cadinol, *α*-cadinol, and *α*-humulene present were common to this study and results from other works (Table 5).

For the fruit pulp essential oils, Ç-sitosterol, a plant steroidal hormone necessary for some physiological processes such as cell elongation and cellulose biosynthesis [44], was identified as the most abundant component. Campesterol, another phytosterol with similar functions as sitosterol, was also identified in the fruit essential oils. In general, however, majority of the components identified in the fruit essential oils were aliphatic compounds made up of esters, alkanes, alcohols, aldehydes, and fatty acids. 2-Hexenoic acid, methyl ester (or methyl-2-hexenoate) which was present in this study as the 2^nd^ most abundant constituent, was present in the pulp essential oils from other locations [28, 30, 33, 34]. The levels were however different. Variations in essential oil composition from the same plant organ may be due to a variety of factors. Seasonal variations in chemical composition of essential oils have been reported in the literature. This difference in chemical composition due to seasonal variations has been observed to affect the bioactivity of the essential oils as well [45, 46]. Other reasons for differences in the chemical composition of essential oils from the same plant organ include environmental and genetic factors, geographical variations, chemotypic diversity, plant maturation stage, and nutritional status of the plant [46–49]. Thus, two plants of the same species but different chemotypes will produce essential oils with variations in chemical composition. The differences in essential oil compositions of *Annona muricata* leaf and fruit pulp in this and other works may be attributed to one or any combination of these elements.

The antioxidant potential of the leaf and fruit pulp essential oils, as evaluated from the phosphomolybdenum, DPPH scavenging, and hydrogen peroxide assays, revealed promising antioxidant properties (Tables 3 and 4). The mechanism of antioxidant activity includes prevention of the formation of the initiator radical and termination of radicals in the chain propagation stage or via indirect routes such as enhancement of the activities of enzymes that mop up reactive species or induction in the expression of these enzymes [18, 50]. Because essential oils consist of a number of different compounds, the antioxidant activity observed is usually the synergistic effect of the various constituents [18]. In the DPPH radical scavenging assay, a dose-dependent inhibition of the DPPH radical was observed in essential oil and standard ascorbic acid treatments. The IC_50_ values obtained for the leaf and fruit pulp essential oils indicate a good inhibition of the DPPH radical. In the hydrogen peroxide scavenging assay, the leaf essential oil showed superior activity to the pulp essential oil. The activity of both essential oils could however be described as average as the percent inhibition of hydrogen peroxide was less than 50% for both oils.

Both essential oils, however, displayed very good total antioxidant activity, as seen in Table 3. Both oils had high levels of phenolic content, and this may have contributed to the antioxidant activities observed. It has been postulated that phenolic compounds possess high reactivity towards peroxyl radicals via a formal hydrogen atom transfer, and this is the basis of their antioxidant activity [18, 51]. Phenolic compounds are therefore categorized as chain breaking antioxidants [52]. Many reports on the antioxidant potential of essential oils exist [13], and this report further confirms the importance of essential oils as antioxidative agents. Since reactive oxygen species cause serious damage to nucleic acids, proteins, and lipids, the impressive antioxidant activity of the essential oils from *Annona muricata* suggests possible use as natural antioxidant source in food additives and animal feed formulation.

## 5. Conclusion

Sesquiterpenes were the major constituents of the leaf essential oils of *Annona muricata* from Ghana, whereas the fruit pulp essential oil consisted of aliphatic compounds and sterols. The most abundant compound in leaf essential oil was *δ*-cadinene. In the fruit pulp essential oil, Ç-sitosterol was most abundant. Both essential oils displayed promising antioxidant activities and suggests potential application in food processing and preservation as well as cosmetic and pharmaceutical industries.

## Figures and Tables

**Table 1:** Composition of leaf essential oil of *Annona muricata* as determined by GC-MS with tentative identified compounds.

No.	RT	SI	Compound	% composition
1	7.16	87	*α*-Pinene, (D)-	0.04
2	8.87	96	Limonene, (D)-	0.25
3	11.59	98	Methyl (9E)-9-octadecen-12-ynoate	0.36
4	13.06	89	6-Methyloctadecane	0.21
5	13.62	90	*δ*-EIemene	1.04
6	14.21	98	*α*-Copaene	4.21
7	14.39	99	*β*-Elemene	0.18
8	14.65	95	*α*-Muurolene	10.64
9	14.65	91	Andrographolide	6.51
10	14.84	98	Ledene oxide (II)	6.29
11	14.84	98	*β*-Caryophyllene	5.57
12	15.08	92	*α*-Acorenol	0.06
13	15.14	83	*β*-cis-farnesene	2.72
14	15.30	97	*α*-Humulene	1.44
15	15.37	97	*γ*-Muurolene	0.21
16	15.52	98	*α*-Selinene	1.13
17	15.74	91	4-epi-cubedol	0.88
18	16.01	96	Caryophyllene oxide	1.15
19	16.09	97	*δ*-Cadinene	22.58
20	16.80	97	Cubenol	1.94
21	16.91	94	2-Hexadecen-1-ol, 3,7,11,15-tetramethyl	0.39
22	17.75	97	*α*-Cadinol	5.65
23	19.30	96	Methyl 10,12-pentacosadiynoate	1.99
24	19.63	88	*β*-Viternene	0.25
25	19.73	91	Cedren-13-ol	0.12
26	20.07	95	Gitoxigenin	1.37
27	20.14	96	16-Octadecenoic acid, methyl ester	0.26
28	20.26	95	3-(Octadecyloxy) propyl ester	5.57
29	22.35	94	6-epi-shyobunol	5.17
30	25.57	99	*τ*-Cadinol	6.47
31	27.27	90	Octadecane	5.33
			Terpenes	50.26
			Oxygenated terpenes (terpenoids)	34.24
			Esters	8.18
			Alkanes	5.54
			Sterol	1.37
			Alcohol	0.39
			Total	99.98

No., compound number in order of elution; SI, Similarity Index (library search of purity value of a compound); RT, retention time in minutes.

**Table 2:** Composition of fruit pulp essential oil of *Annona muricata* as determined by GC-MS with tentative identified compounds.

No.	RT	SI	Compound	% composition
1	6.51	80	Nonane	1.74
2	6.96	98	Hexanoic acid methyl ester	1.86
3	7.74	96	2-Hexenoic acid, methyl ester	10.27
4	8.11	94	Pentanoic acid, 2-hydroxy-4-methyl-, methyl ester	0.03
5	8.18	98	Pentanoic acid, 2-hydroxy-3-methyl-, methyl ester	0.42
6	9.35	94	2,6,8-Trimethylbicyclo[4.2.0]oct-2-ene-1,8-diol	0.23
7	10.02	99	1,6-Octadien-3-ol, 3,7-dimethyl-	0.13
8	11.12	94	2-Octenoic acid, methyl ester, (E)-	0.48
9	11.58	92	Dodecane	0.59
10	12.00	94	Phenylacetaldehyde	1.71
11	13.05	91	Tridecane	0.43
12	14.31	97	2-Propenoic acid, 3-phenyl-, methyl ester	8.67
13	14.83	98	*β*-Caryophyllene	1.77
14	15.30	96	4,5-di-epi-Aristolochene	0.35
15	15.72	93	Falcarinol	0.18
16	16.05	99	*β*-Guaiene	0.17
17	20.54	94	Hexadecanoic acid, methyl ester	0.33
18	22.26	94	Glyceryl 2-laurate	1.52
19	22.31	99	9-Octadecenoic acid, methyl ester, (E)-	2.18
20	25.07	97	8-Octadecanoic acid	2.20
21	25.08	99	2-Hydroxy-1-(hydroxymethyl) ethyl ester	13.48
22	25.58	96	Ç-Sitosterol	19.82
23	26.44	94	9-Octadecanoic acid	4.59
24	26.44	96	Pentadecanoic acid, 13-methyl-, methyl ester	0.42
25	26.93	80	2,3-Dihydroxypropyl elaidate	3.81
26	27.02	96	Squalene	1.74
27	27.28	97	*α*-Sitosterol	3.04
28	27.28	99	Oleic acid	2.00
29	27.63	87	Octadecane	2.03
30	29.81	91	Campesterol	2.19
31	30.62	96	ϒ-Sitosterol	4.96
32	32.44	99	Oleyl oleate	2.53
33	32.65	86	Nonadecatriene-5,14-diol	4.12
			Esters	46.00
			Sterols	30.01
			Terpenes	4.03
			Alcohols	4.66
			Alkanes	4.79
			Aldehyde	1.71
			Carboxylic acid	8.79
			Total	99.99

No., compound number in order of elution; SI, Similarity Index (library search of purity value of a compound); RT, retention time in minutes.

**Table 3:** Total antioxidant capacity (TAC) and total phenolic content (TPC) of fruit pulp and leaf essential oils.

Sample	TAC^*∗*^ gAAE/100 g	TPC^*∗∗*^ gGAE/100 g
Pulp essential oil	49.03 ± 0.48^a^	3.98 ± 0.60^b^
Leaf essential oil	50.88 ± 0.50^a^	4.38 ± 0.42^b^

Columns with different letters indicate significant difference (*P* < 0.05, Sidak's multiple comparison test). ^*∗*^TAC expressed in gram ascorbic acid equivalent per 100 g of sample (gAAE/100 g). ^*∗∗*^TPC expressed in gram gallic acid equivalent per 100 g of sample (gGAE/100 g).

**Table 4:** DPPH free radical and hydrogen peroxide scavenging activities of essential oils and hydrosols from leaf and fruit pulp of *Annona muricata*.

Sample	IC_50_ (*μ*g/mL)^*∗*^	% H_2_O_2_ scavenged^*∗∗*^
Pulp essential oil	512.0 ± 5.1	24.38 ± 0.21
Leaf essential oil	244.8 ± 3.2	31.80 ± 0.51
Ascorbic acid	21.3 ± 3.2	ND
Gallic acid	ND	95.00 ± 1.12^*∗∗∗*^

^*∗*^IC_50_, concentration of extract required to scavenge 50% of DPPH radicals. ^*∗∗*^% H_2_O_2_ scavenged was determined at 1 mg/mL for essential oils. ^*∗∗∗*^For gallic acid, % H_2_O_2_ scavenged was determined at 50 *μ*g/mL.

**Table 5:** Comparison of leaf and fruit pulp essential oil composition from different studies.

Source	Location	Constituents (% composition)
*Leaves*		
Present findings	Ghana	*δ*-Cadinene (22.58), *α*-muurolene (10.64), andrographolide (6.51), *τ*-cadinol (6.47), ledene oxide (II) (6.29), *α*-cadinol (5.65), *β*-caryophyllene (5.57),
[33]	Cote d'Ivoire	*β*-Caryophyllene (31.4), *δ*-cadinene (6.7), *α*-muurolene (5.5), (E)-2-hexenol (4.8), *τ*-cadinol (4.3)
[29]	Benin	*β*-Caryophyllene (13.6), *δ*-cadinene (9.1), epi-*α*-cadinol (8.4), *α*-cadinol (8.3), isocaryophyllene (7.5)
[32]	Nigeria	(E)-Caryophyllene (38.9), eugenol (30.2), *δ*-cadinene (6.0), caryophyllene oxide (5.0), *α*-humulene (4.3)
[35]	Vietnam	*β*-Pinene (20.6), germacrene D (18.1), *p-*mentha-2,4(8)-diene (9.8), *α*-pinene (9.4), *β*-elemene (9.1)
[27]	Cameroon	*β*-Caryophyllene (40), *β*-elemene (14.4), *α*-santalene (9.5), (Z)-hex-3-enol (5.2), *δ*-cardinene (4.8)
[26]	Egypt	Bicycloelemene (23.6), limonene (16.6), *β*-pinene (14.3), *α*-fenchene (6.6), *α*-pinene (5.4)

*Fruit pulp*		
Present findings	Ghana	Ç-Sitosterol (19.82), 2-hydroxy-1-(hydroxymethyl) ethyl ester (13.48), 2-hexenoic acid, methyl ester (10.27), 2-propenoic acid, 3-phenyl-, methyl ester (8.67), ϒ-sitosterol (4.96)
[36]	Malaysia	Methyl (E)-2-butenoate (19.70), Methyl (E)-2-hexenoate (18.40), (Z)-3-hexenol (9.7), linalool (9.30), methyl 2-hydroxyhexanoate (5.2)
[33]	Cote d'Ivoire	Methyl (E)-2-hexenoate (39.8), 3-pyridinocarbonylhydrazide (7.8) methyl hexanoate (5.4), methyl (E)-2-butenoate (4.8), 2,3-dihydrobenzofuran (4.5)
[34]	Cuba	Methyl 3-phenyl-2-propenoate (10.6), hexadecanoic acid (9.7), methyl (E)-2-hexenoate (8.8), methyl 2-hydroxy-4-methylvalerate (7.2), linalool (2.9)
[30]	Sri Lanka	Methyl hexanoate (30.95), methyl (E)-hex-2-enoate (26.70), trans-*β*-farnesene (6.45), dichloromethane (5.72), methyl but-2-enoate (4.75)
[28]	Cameroon	2-Hexenoic acid methyl ester (23.9), *β*-caryophyllene (12.7), 1,8-cineole (9.9), 2-hexenoic acid ethyl ester (8.6), linalool (7.8)

## Data Availability

All data generated or analyzed during this study are included in this published article.
